# Acceptability and completeness of vaccination schedules recommended for transgender women and *travestis* in the city of São Paulo, Brazil: a cross-sectional study, 2019-2020

**DOI:** 10.1590/S2237-96222024v33e2024341.especial.en

**Published:** 2025-01-13

**Authors:** Cristina Langkammer Martins, Katia Cristina Bassichetto, Maria A. S. M. Veras

**Affiliations:** 1 Centro de Referência e Treinamento DST/Aids-SP, São Paulo, SP, Brasil; 2 Faculdade de Ciências Médicas da Santa Casa de São Paulo, São Paulo, SP, Brasil

**Keywords:** Personas Transgénero, Vacunación, Hepatitis A, Hepatitis B, Virus del Papiloma Humano, Transgender People, Vaccination, Hepatitis A, Hepatitis B, Human Papillomavirus

## Abstract

**Objective:**

To assess adherence to and completeness of vaccination schedules against human papillomavirus (HPV) and hepatitis A and B among transgender women and *travestis* in São Paulo, capital city of São Paulo state.

**Methods:**

This was a secondary data analysis of the multicenter TransOdara study. Data were collected from 403 transgender women and *travestis* aged 18 years or older, recruited through respondent-driven sampling between December 2019 and October 2020.

**Results:**

High adherence to vaccines was observed (88.8%), but completeness of the analyzed vaccination schedules was low: 12% (95%CI 8.0; 17.3) for hepatitis A, 7.2% (95%CI 3.5; 12.8) for hepatitis B and 8.1% (95%CI 3.0; 16.6) for HPV, with no statistically significant differences between them.

**Conclusion:**

Despite high adherence, the low completion of vaccination schedules highlighted the need for diversified strategies to improve vaccination coverage and reduce the prevalence of vaccine-preventable sexually transmitted infections in this population.

## Study contributions

**Main results:**

The high adherence of participants to receiving immunizations offered against vaccine-preventable sexually transmitted infections did not result in high rates of completeness for the hepatitis A, human papillomavirus and hepatitis B vaccination schedules.

**Implications for services:**

Welcoming and inclusive environments must be established to enhance access to healthcare services for transgender women and *travestis*. Professional training is needed to improve the quality of care.

**Perspectives:**

Implementing strategies such as active outreach for individuals overdue for vaccination doses and raising awareness within the transgender population, can contribute to greater adherence and completeness of the vaccination schedules recommended for this population.

## RESUMEN

**Objetivo:**

Evaluar la adhesión y la completitud de los esquemas de vacunación contra el virus del papiloma humano (VPH) y las hepatitis A y B en mujeres trans y travestis en el municipio de São Paulo, Brasil.

**Métodos:**

Se trata de un análisis secundario de datos del estudio multicéntrico TransOdara. Se utilizaron datos de 403 mujeres trans y travestis mayores de 18 años, reclutadas mediante el método de muestreo dirigido por los propios participantes (respondent-driven sampling), entre diciembre de 2019 y octubre de 2020.

**Resultados:**

Se observó una alta adhesión a las vacunas (88,8%), pero una baja completitud de los esquemas de vacunación analizados: 12% (IC95% 8,0;17,3) para la hepatitis A, 7,2% (IC95% 3,5;12,8) para la hepatitis B y 8,1% (IC95% 3,0;16,6) para el VPH, sin diferencias estadísticamente significativas entre ellos.

**Conclusión:**

A pesar de la alta adhesión, la baja completitud de los esquemas de vacunación indicó la necesidad de estrategias diversificadas para mejorar la cobertura vacunal y reducir la prevalencia de infecciones de transmisión sexual prevenibles mediante inmunización en esta población.

## INTRODUCTION

Sexually transmitted infections (STIs) affect more than 1 million people a day worldwide.^1^ Human papillomavirus (HPV) infection is among the most common STIs, with estimates suggesting that 50% to 100% of the sexually active population will contract the virus at some point in their lives.^2^ It has been estimated that one in three men aged 15 years and older is infected with at least one subtype of HPV.^3^ The incidence of more than 100 million per year was estimated for hepatitis A virus infection. This number corresponded to more than 296 million for the hepatitis B virus.^1,4^ In Brazil, between 2000 and 2021, 168,175 cases of hepatitis A were reported, with a higher concentration in the Northeast region (30%), alongside 264,640 cases of hepatitis B.^5^

HPV and hepatitis A and B infections are frequently contracted through unprotected sexual practices.^6^ These infections are preventable through highly effective vaccines if administered at the appropriate time and in the doses recommended by the National Immunization Program (*Programa Nacional de Imunização* - PNI).^7^ In Brazil, since 2014, the HPV vaccine has been recommended for girls and boys aged 9 to 14 years and for people living with HIV/AIDS up to 45 years of age.^8^ The hepatitis B vaccine has been universally offered since 1998, and is administered within the first hours of life. The hepatitis A vaccine is recommended for children up to 4 years of age and adults with pre-existing health conditions. The PNI lacks specific guidelines for vaccinating men who have sex with men and the transgender population.^6^

Prevention is the main tool for mitigating STI-related harm in both the general population and the transgender population. The combined prevention strategy aims to reduce the prevalence of STIs in high-risk populations, specifically including immunization against HPV and hepatitis B.^10^ Although the hepatitis A vaccine is not universally recommended for the transgender population, it has been incorporated in the adult vaccination schedule in the state of São Paulo.^11^

The absence of updated data on the prevalence and incidence of STIs in the transgender population makes it difficult to understand their magnitude and to develop appropriate health policies aimed at their specific needs. It is crucial to assess how trans women and *travestis* benefit from the immunization program and whether they are able to access vaccines as a tool for preventing STIs. The objective of this study was to evaluate the acceptability and completeness of vaccination schedules for the hepatitis A, hepatitis B and HPV, included in the adult vaccination schedule, among trans women and *travestis* in the city of São Paulo. The reasons for vaccine refusal were also investigated.

## METHODS

Secondary data from the TransOdara, a multicenter cross-sectional study, were used. This study investigated the prevalence of infections including syphilis, HIV, hepatitis A, B and C, gonorrhea, chlamydia and HPV among 1,317 trans women and *travestis*. aged 18 years and older. They were recruited across five Brazilian capitals between December 2019 and July 2021. The study also investigated the effectiveness of the point-of-care approach, which consisted of offering treatment for diagnosed STIs and preventive interventions, including HPV and hepatitis A and B vaccines, on the same day they took part in the research.

The sample size was calculated to estimate the prevalence of active syphilis, defined as titers greater than 1:8 of the venereal disease research laboratory (VDRL). A suitable method for populations of unknown size was used, taking into consideration the characteristics of the cities.^12^

In this study, data from 403 trans and *travesti* women recruited in the city of São Paulo between December 2019 and October 2020 were analyzed. The respondent - driven sampling method, considered effective for reaching hard-to-reach populations^13^ was employed. It was also used in other studies in Brazil.^14^ This method involved the initial selection of “seeds”, i.e., trans people with extensive social networks who could refer up to six potential participants. Referred individuals received numbered coupons to trace the origin and sequence of recruitment.

After confirming eligibility requirements and signing the free and informed consent form, participants provided biological sample, answered structured questionnaires, and underwent nursing and medical consultations. Data collection and clinical evaluation were conducted using the following forms: acceptability of collection and pre-consultation procedures, acceptability of collection and post-consultation procedures, clinical evaluation and follow-up, and laboratory evaluation. Based on test results, participants received treatments prescriptions and vaccines available through the Brazilian National Health System as recommended by the PNI and prior STI history and treatment. A “peer navigator”, also a trans woman, assisted monitor the participants, especially during the vaccination process. Most participants were not registered at the study site, the STI/AIDS Reference and Training Center, and were assigned an identifier number for easier record-keeping and follow-up.

At the end of their participation in the study, each volunteer received financial support to cover transportation and meal expenses incurred to attend the study site.

In order to characterize the socioeconomic and demographic variables of this study, the following independent variables were analyzed: age, divided into age groups (in years: 18-19, 20-29, 30-39, ≥40); employment status (unemployed, work on the books, self-employed, work off the books, occasional/temporary jobs, retired/on benefits), primary occupation being as sex worker (yes, no); education (in years: ≤8, 9-12, ≥13); housing status (living in a rented/owned house or apartment, unstable housing [shelter/institution/homeless or without fixed address, in a boarding house/brothel/invasions or occupied property], temporarily living with family/friends); religion (no religion, Catholic, Afro-Brazilian [Umbanda, Candomblé], Evangelical/Protestantism, Spiritualism , Oriental [Buddhism/Hinduism], Judaism, refused to answer), ever had sex in exchange for money, goods, drugs or housing (yes, no, did not answer), gender identity (transgender woman, *travesti*, woman, androgynous/ambiguous gender/non-binary) and sexual orientation (heterosexual, bisexual, pansexual, homosexual/gay or lesbian, I do not know, asexual).

The dependent variables were adherence to and completeness of proposed vaccination schedules. Adherence was categorized as “yes”, “no” and “unsure”. Completeness (defined as “receiving the required doses”) was categorized as “yes” and “no”.

In order to calculate the proportions of vaccination adherence, TransOdara data available on Research Electronic Data Capture (REDCap) platform, ^15^ were used. These data were obtained by filling out the post-consultation procedure acceptability form, which included a question about interest in receiving the HPV and hepatitis A and B vaccines. Participants were informed about the required doses for each vaccine, administration sites, and the need for follow-up to complete the indicated schedule.

The indicators selected to measure the proportions of adherence to the vaccines of interest included: proportion of participants who agreed to be vaccinated if indicated; proportion of participants who agreed to receive an injection in the arm during the visit and subsequent doses between one to six months later; and proportion of participants available to return to receive subsequent doses of the recommended vaccines. Two participants did not respond these last two questions when they were interviewed.

Reasons for vaccine refusal were analyzed through open-ended questionnaire responses, and the most frequently cited reasons were reported as percentages.

In order to assess completeness, the nurse from the vaccination room and research team accessed the electronic medical records to verify the participants’ return for vaccine doses. The information was recorded in REDCap to compose the research database. The proportions of vaccine completeness were calculated based on the proportion of participants who received the recommended doses: HPV vaccine (1^st^, 2^nd^ and 3^rd^ doses), hepatitis A vaccine (1^st^ and 2^nd^ doses) and hepatitis B vaccine (1^st^, 2^nd^ and 3^rd^ doses), considering only those indicated for each vaccine.

The recommendations for immunizations followed the criteria of the PNI vaccination schedule, taking into account immunization history, age, sexual practice and HIV serological status. This explained the variation in the number of participants eligible for each type of vaccine. Recruitment was completed in October 2020, with follow-ups at the vaccination clinic considered up to August 2021.

For the hepatitis A vaccine, five participants were referred to initiate vaccination. Of these, three did not receive the first dose for unspecified reasons. Two did not begin the vaccination schedule because existing records indicated prior doses of the vaccine had already been administered before the study.

Regarding the hepatitis B vaccine, three participants were referred for vaccination; however, two withdrew without justification upon arriving at the vaccination clinic. One participant did not initiate the schedule due to prior completion of the vaccine series, as documented in the electronic medical records. One participant initiated the series but, upon returning for the second dose, it was confirmed that the series had already been completed before the study.

During the COVID-19 pandemic, participant recruitment in São Paulo, conducted at a reference healthcare facility for HIV/AIDS and STIs that remained operational throughout the pandemic, was interrupted after reaching half of the planned sample size. A four-month pause was implemented to comply with restrictive measures. However, the planned sample size was ultimately achieved as recruitment resumed afterward.

The variables were described using absolute and relative frequencies, stratified as previously specified. The 95% confidence interval (95%CI) for vaccine completion rates was calculated for each vaccine. Respondent-driven sampling weights were not applied in the TransOdara analysis due to existing studies contraindicating their use. All statistical analyses were conducted using Stata 14.1.

The research was approved by the Research Ethics Committee of Santa Casa de Misericórdia de São Paulo through opinion No. 3,126,815 of 01/30/2019; certificate of presentation of ethical appraisal 05585518.7.0000.5479.

## RESULTS

The TransOdara study in São Paulo involved 403 transgender women and *travestis*, with an average age of 33.9 years. Of the total, 38.0% were between 20 and 29 years old, 25.0% were unemployed and 21.6% identified as sex workers, while 50.2% reported having exchanged sex for money, drugs, housing or other goods. The majority of participants (69%) had between 9 and 12 years of study, and 72% lived in their own or rented house or apartment. 37% of the participants reported no religious affiliation. Most participants (66.3%) identified as trans women, and 85.6% were heterosexual (Table 1).

**Table 1:** Socioeconomic and demographic characteristics of participants in the TransOdara Project, municipality of São Paulo, Brazil, 2019-2020 (n=403)

Variables	n (%)
**Age group (years)**	
18-19	7 (1.7)
20-29	153 (38.0)
30-39	126 (31.3)
≥40	117 (29.0)
**Employment status** ^a^	
Unemployed	69 (25.0)
Work on the books	62 (22.5)
Self-employed	55 (19.9)
Work off the books	41 (14.8)
Engage in occasional/temporary work	39 (14.1)
Retired/on social benefits	10 (3.6)
**Primary occupation in sex work**	
Yes	87 (21.6)
No	316 (78.4)
**Education (years)**	
≤8	67 (16.6)
9-12	280 (69.5)
≥13	56 (13.9)
**Housing situation**	
Rent/own a house or apartment	291 (72.2)
Unstable housing	64 (15.9)
Temporarily live with family/friends	48 (11.9)
**Religion**	
No religion	149 (37.1)
Catholic	91 (22.5)
Afro-Brazilian (Umbanda or Candomblé)	68 (16.8)
Evangelical/Protestant	52 (12.9)
Spiritist	35 (8.7)
Eastern/Buddhism/Hinduism	4 (1.0)
Jewish	2 (0.5)
Refused to answer	2 (0.5)
**Had sex in exchange for money?**	
No	90 (22.3)
Yes	203 (50.2)
Did not answer	111 (27.5)
**Gender identity**	
transgender woman	267 (66.3)
Transvestite	95 (23.6)
Woman	39 (9.7)
Androgynous/gender ambiguous/non-binary	2 (0.4)
**Sexual orientation**	
Heterosexual	346 (85.6)
Bisexual	22 (3.4)
Pansexual	17 (4.2)
Homosexual, gay or lesbian	16 (4.0)
Unsure	2 (0.5)
Asexual	1 (0.2)

a) One hundred and twenty-seven participants did not disclose their employment status at the time of the survey.

A total of 88.8% of participants were interested in receiving the recommended vaccines. Of these, 96.6% stated they would accept an intramuscular injection in the arm on the same day of their visit and, followed by another vaccine dose between one and six months later. They also stated that they would be available to return and receive the remaining doses (Table 2).

**Table 2:** Prior interest in receiving intramuscular vaccines and completing recommended vaccination schedules, municipality of São Paulo, Brazil, 2019-2020 (n=403)

Interest	Yes (%)	No (%)	Unsure (%)	Total
Willingness to receive the recommended vaccines	358 (88.8)	44 (10.9)	1 (0.2)	403
Acceptance of receiving an injection in the arm on the survey day and, follow-up doses within one to six months	346 (96.6)	05 (1.4)	5 (1.4)	356^a^
Willingness to return for subsequent vaccine doses	346 (96.6)	05 (1.4)	5 (1.4)	356^a^

a) 2 participants did not respond to questions about prior acceptance of receiving an injection in the arm on the survey day and follow-up doses within one to six months, as well as their willingness to return for subsequent vaccinations.

The referral to the vaccination room was refused by 44 participants, regardless of the recommended vaccination schedule. The reasons for refusal were also documented, with the most commonly reported being: having an up-to-date or complete vaccination booklet, fear or aversion to needles, being in a hurry or preferring to receive the vaccine at another healthcare center (Table 3).

**Table 3:** Reasons provided by participants for refusing the vaccines recommended by the TransOdara project professionals, municipality of São Paulo, Brazil, 2019-2020 (n=44)

Reason for refusing recommended vaccines	n
Complete vaccination booklet	27
Fear of needles	7
Lack of time	3
Preference for another healthcare service	3
Lack of interest	1
Residence far from the place where the first dose was received	1
Aversion to medication use	1
Preference for evaluation by an infectious disease specialist	1

It could be seen that 77.2% (95%CI 71.7;82.1) received the first dose of the hepatitis A vaccine, 70.9% (95% CI 64.0;77.2) received the hepatitis B vaccine, and 88.2% (95% CI 79.4;94.2) received the HPV vaccine. The proportion of refusal of the first dose of the HPV vaccine (11.8%, 95% CI 5.8;20.6) was significantly lower than that observed for the hepatitis B vaccine (27.5%, 95% CI 21.4;34.4). The completeness rates for the vaccination schedules were 12.0% (95%CI 8.0; 17.3) for the hepatitis A vaccine, 7.2% (95%CI 3.5;12.8) for the hepatitis B vaccine and 8.1% (95%CI 3.0;16.6) for the HPV vaccine, with no statistically significant difference observed among them.

Some participants who initially agreed to receive the recommended vaccination schedules refused administration upon arriving at the vaccinatio**n** clinic, with refusal rates of 20.8% for the hepatitis A vaccine, 27.5% for the hepatitis B vaccine, and 11.8% for the HPV vaccine (Table 4).

**Table 4:** Indication, number and 95% confidence interval (95%CI) of doses received for vaccines against hepatitis A, hepatitis B and human papillomavirus (HPV) by participants, municipality of São Paulo, Brazil 2019-2021

Dose	Hepatitis A (n=268)		Hepatitis B (n=196)		HPV (n=85)	
	n	% (95%CI)	n	% (95%CI)	n	% (95%CI)
**First**						
Accepted	207	77.2 (71.7;82.1)	139	70.9 (64.0;77.2)	75	88.2 (79.4;94.2)
Refused	56	20.8 (16.2;26.3)	54	27.5 (21.4;34.4)	10	11.8 (5.8;20.6)
Other situation	5	1.9 (0.6;4.3)	3	1.5 (0.3;4.4)	-	-
**Second**						
Administered	25	12.0 (8.0;17.3)	19	13.7 (8.4;20.5)	10	13.3 (6.6;23.2)
Did not attend	182	86.1 (82.7;92.0)	119	85.6 (78.7;91.0)	65	86.7 (76.8;93.4)
Other situation	-	-	1	0.7 (0.01;3.9)	-	-
**Third**						
Administered	-	-	10	7.2 (3.5;12.8)	6	8.1 (3.0;16.6)
Did not attend	-	-	9	6.5 (3.0;11.9)	4	5.3 (1.5;13.1)
Other situation	-	-	-	-	-	-

## DISCUSSION

High adherence to HPV and hepatitis A and B vaccines was observed yet low completion rates of vaccination schedules persisted, even after participants were informed by the research team about the benefits, administration routes, and timing of each vaccine. The lack of specific studies on vaccine uptake among transgender women and *travestis* as a preventive strategy for sexually transmitted infections (STIs) complicates direct comparisons of the results. Data from this study were compared with those from men who have sex with men (MSM), people living with HIV, and the general population, particularly concerning hepatitis A vaccine completeness.

Analysis of the acceptance of the hepatitis A vaccine among different populations can reveal variables that influence adherence to immunization. In this study, the observed acceptance rate was higher than that recorded among men who have sex with men in Victoria, Australia, which was 62.7%. Such acceptance was measured by receipt of at least one dose of the vaccine.^17^ In this study, the expression of willingness to receive the vaccine was considered as an acceptance criterion.

The study population**s** shared significant similarities in terms of sexual vulnerabilities and vaccine recommendations but differed in adherence proportions. This discrepancy could be explained by the difference in mean age: 33.9 years in this study versus 29 years in Victoria. This age difference reflected varying levels of awareness regarding immunization and health priorities. While vaccination in Victoria was offered based on prior serological status, this study assessed willingness to receive the vaccine among all participants.

Adherence to the HPV vaccine in this study was 88.8%, a positive result that indicated the participants’ intention to use the vaccine as a strategy to prevent STIs. This figure was close to the 87.0% found in Mexico City, which included an adult population, with 9.3% trans women.^18^A similar value of 90.0% was observed among sex workers in Teresina, Piauí state,^19^ for the hepatitis B vaccine. The high rates of adherence to the HPV and hepatitis B vaccination schedules seemed related to participants’ maturity, whose average age ranged from 31.9 to 33.9 years. This suggested that maturity may influence awareness of sexual health and acceptance of vaccines, in addition to promoting the appreciation for prevention and health care opportunities.

Regarding the hepatitis B vaccine, the adherence in this study was higher than that observed in Paris, which included high-risk populations such as men who have sex with men, people who inject drugs and trans women. Among those eligible who were neither vaccinated nor infected, acceptability to receive the first dose was 53.0% among trans women.^20^

Hard-to-reach populations, trans women and *travestis*, sex workers and men who have sex with men, are more vulnerable due to sexual practices and, consequently, have a higher risk of contracting curable STIs. Therefore, it is not enough to have vaccines available in health services aimed at these population groups. To improve adherence rates for vaccination schedules, strategies such as active search must be incorporated

The completeness of the HPV vaccination schedule in this study showed intermediate results compared to a sample of men who have sex with men and trans women from two cities in the United States, where 4.6% reported having received the three doses, mainly in private services.^21^ In contrast, a small national sample of young adults identified as transgender in the United States showed completeness above 60%.^22^ Similarities in characteristics related to vaccine indication, age group and high level of education were insufficient to ensure that the participants in this sample completed the vaccination schedule. Economic barriers, highlighted in the studies conducted in that country, show that the greatest access occurred in private clinics and that healthcare professionals lacked incentives. In this study, vaccination was offered in public healthcare services, and low completion rates may have been associated with financial difficulties, such as affording public transportation costs to return for subsequent doses, as 25% of the population reported being unemployed at recruitment.

The provision of the hepatitis A vaccine has not ensured high completeness rates of the vaccination schedule, even when offered free of charge to people living with HIV in Brazil and some Central and Western Europe countries.^23^ In France, 54.2% of people living with HIV and recommended for vaccination were immunized.^24^ In the United Kingdom, the vaccine is also free and recommended for high-risk adults, including men who have sex with men. Among the general British population, the completeness of the vaccination schedule for hepatitis A reached 11%.^25^

Low completion of the hepatitis B vaccination schedule was observed in Paris, where no participant returned for the third dose.^20^ In Teresina, sex workers achieved a 26.3% completion rate due to a scheduling strategy that accommodated participants’ availability.^19^ Similar scheduling strategies were adopted in Goiânia, Goiás state, resulting in completion rates of 55.3% and 41.5% for two different schedules (a conventional three-dose schedule and an accelerated four-dose schedule).^26^ Shorter vaccination schedules have been suggested to improve adherence, especially among hard-to-reach populations.

The COVID-19 pandemic had a significant impact on the completeness of vaccination schedules, especially among vulnerable populations such as sexual minorities, both due to financial issues and the distancing measures proposed by the state government.^27^ Online recruitment of trans individuals in Brazil showed that 59.0% reported a high impact from social distancing measures, with 54.7% indicating that the economic situation was the most affected.^28^ The main challenges faced were job retention and salary reduction. Despite continued vaccination services, schedule completion was negatively affected.

Peer navigator strategy has proven effective in improving vaccine adherence, promoting self-care, and increasing treatment engagement, especially among vulnerable groups. This model facilitated access to health services and disseminate knowledge about preventive practices against STIs. The effectiveness of this approach was observed in São Paulo, where 94.4% of participants recommended peer navigator strategies for antiretroviral treatment adherence among people living with HIV/AIDS.^29^

This study did not include the presence of a peer navigator to accompany participants on subsequent visits to the vaccination room, financial resources to cover the participants’ transportation costs to return to the service, or active search strategies to locate those who missed subsequent doses. It was not possible to verify whether participants received additional doses at other health services within the Brazilian National Health System.

This study demonstrated significant strengths, such as achieving the expected sample size despite disruptions caused by the COVID-19 pandemic. Participants expressed satisfaction with accessing vaccines recommended for STI prevention.

Despite high adherence to receiving vaccines, low completeness rate should draw the attention of immunization policymakers. Taking into consideration the barriers trans people face in accessing health services and the increased risks for vaccine-preventable STIs,^30^ it is essential to raise awareness among health professionals who serve this population. Innovating strategies to expand access to services is also crucial, including implementing new care methodologies that promote the inclusion of this population in routine healthcare services.

One of the main challenges in healthcare is providing continuous education to professionals serving transgender individuals, aiming to raise awareness about the realities and living conditions of this population. This is essential to reduce resistance, expand access, and ensure dignified and respectful care. Educational initiatives targeting the transgender population are also critical to encourage adherence to and completeness of recommended vaccination schedules. A concerted effort by health authorities is needed to increase the availability of vaccines for STI prevention and to inform and raise awareness among healthcare professionals about these preventive measures.
