# Supplementary material for: Acceptability and completeness of vaccination schedules recommended for transgender women and travestis in the city of São Paulo, Brazil: a cross-sectional study, 2019-2020
Source: Epidemiol Serv Saude. 2025 Jan 13;33(spe1):e2024341. doi: 10.1590/S2237-96222024v33e2024341.especial.en (PMC11734595; doi:10.1590/S2237-96222024v33e2024341.especial.en)
Supplement: Translation

# Translation: Aceitabilidade e conclusão dos esquemas vacinais indicados para mulheres trans e travestis na cidade de São Paulo: estudo transversal, 2019-2020

**DOI:** 10.1590/S2237-96222024v33e2024341.especial.pt

**Authors:** Cristina Langkammer Martins, Katia Cristina Bassichetto, Maria A. S. M. Veras

**Affiliations:** 1 Centro de Referência e Treinamento DST/Aids, São Paulo, SP, Brasil; 2 Faculdade de Ciências Médicas da Santa Casa de São Paulo, São Paulo, SP, Brasil

**Keywords:** Pessoas Transgênero, Vacinação, Hepatite A, Hepatite B, Papilomavírus Humano

## Abstract

**Objetivo:**

Avaliar a adesão e a completude dos esquemas vacinais contra papilomavírus humano (*human papillomavirus*, HPV) e hepatites A e B em mulheres trans e travestis no município de São Paulo.

**Métodos:**

Trata-se da análise secundária de dados do estudo multicêntrico TransOdara. Utilizaram-se dados de 403 mulheres trans e travestis maiores de 18 anos, recrutadas no método *respondente-driven sampling*, entre dezembro de 2019 e outubro de 2020.

**Resultados:**

Observou-se alta adesão às vacinas (88,8%), mas baixa completude dos esquemas vacinais analisados: 12% (IC_95_% 8,0;17,3) para hepatite A, 7,2% (IC_95_% 3,5;12,8) para hepatite B e 8,1% (IC_95_% 3,0;16,6) para HPV, sem diferenças estatisticamente significativas entre eles.

**Conclusão:**

Apesar da alta adesão, a baixa completude dos esquemas vacinais indicou a necessidade de estratégias diversificadas para melhorar a cobertura vacinal e reduzir a prevalência de infecções sexualmente transmissíveis preveníveis por imunização nessa população.

## Contribuições do estudo

**Principais resultados:**

A alta adesão das participantes em receber imunizantes ofertados contra infecções sexualmente transmissíveis imunopreveníveis não resultou em altas proporções de completude de hepatite A, papilomavírus humano e hepatite B.

**Implicações para os serviços:**

Ambientes acolhedores e inclusivos devem ser criados para ampliar o acesso das mulheres trans e travestis aos serviços de saúde. Faz-se necessária a capacitação de profissionais para aprimorar a qualidade da atenção.

**Perspectivas:**

A implementação de ações, como a busca ativa das pessoas em atraso de alguma dose de vacina e a conscientização da população trans, pode contribuir para maior adesão e completude dos esquemas vacinais recomendados para essa população.

## INTRODUÇÃO 

As infecções sexualmente transmissíveis (ISTs) afetam mais de 1 milhão de pessoas por dia em todo o mundo.^1^ A infecção pelo papilomavírus humano (*human papillomavirus*, HPV) é uma das ISTs mais comuns, com previsão de que 50% a 100% da população sexualmente ativa contrairá o vírus em algum momento da vida.^2^ Estimou-se que um a cada três homens com idade superior a 15 anos esteja infectado com pelo menos um subtipo de HPV.^3^ A incidência de mais de 100 milhões por ano foi estimada para as infecções do vírus da hepatite A. Esse número correspondeu a mais de 296 milhões para o vírus da hepatite B.^1,4^ No Brasil, entre 2000 e 2021, foram notificados 168.175 casos de hepatite A, com concentração maior na região Nordeste (30%), e 264.640 casos de hepatite B.^5^

As infecções por HPV e hepatites A e B são frequentemente contraídas por práticas sexuais desprotegidas.^6^ Essas infecções são evitáveis com vacinas que apresentam alta eficácia se administradas no tempo certo e nas doses recomendadas pelo Programa Nacional de Imunização (PNI).^7^ No Brasil, desde 2014, a vacina contra HPV é indicada para meninas e meninos de 9 a 14 anos e para pessoas vivendo com HIV/aids até 45 anos.^8^ A vacina contra hepatite B é oferecida universalmente desde 1998, sendo aplicada nas primeiras horas de vida. A vacina contra hepatite A é recomendada para crianças até 4 anos e adultos com doenças preexistentes.^9^ Não há diretrizes específicas do PNI para vacinar homens que fazem sexo com homens e a população trans.^6^

A prevenção é a principal ferramenta para reduzir os danos relacionados às ISTs, tanto na população em geral quanto na população trans. A estratégia de prevenção combinada visa diminuir ás prevalências de ISTs em populações de risco aumentado, incluindo especificamente a imunização contra HPV e hepatite B.^10^ Embora a vacina contra hepatite A não seja indicada universalmente para a população trans, ela foi incluída no calendário vacinal de adultos no estado de São Paulo.^11^

A falta de dados atualizados sobre a prevalência e incidência de ISTs na população trans dificulta o entendimento da sua magnitude e a formulação de políticas de saúde adequadas para atender às suas necessidades específicas. É importante avaliar o quanto as mulheres trans e travestis se beneficiam do programa de imunização e se elas conseguem acessar vacinas como ferramenta de prevenção de ISTs. O objetivo deste estudo foi avaliar a aceitabilidade e a completude dos esquemas vacinais de hepatite A, hepatite B e HPV, que estão no calendário de adultos, entre mulheres trans e travestis no município de São Paulo. Os motivos para a recusa dessas vacinas também foram investigados.

## MÉTODOS

Foram utilizados dados secundários do estudo transversal, multicêntrico TransOdara. Esse estudo investigou a prevalência de infecção por sífilis, HIV, hepatites A, B e C, gonorreia, clamídia e HPV em 1.317 mulheres trans e travestis maiores de 18 anos. Estas foram recrutadas em cinco capitais brasileiras entre dezembro de 2019 e julho de 2021. O projeto também investigou a efetividade da abordagem “*point of care*”, que consistiu na oferta do tratamento para as ISTs diagnosticadas e dos insumos de prevenção, entre eles as vacinas contra HPV hepatites A e B, no mesmo dia em que participaram da pesquisa.

O tamanho da amostra foi calculado para estimar a prevalência de sífilis ativa, definida por títulos superiores a 1:8 do Venereal Disease Research Laboratory (VDRL). Utilizou-se método adequado para populações com tamanho desconhecido, considerando as características das cidades.^12^

Neste estudo, foram analisados dados de 403 mulheres trans e travestis recrutadas no município de São Paulo entre dezembro de 2019 e outubro de 2020. Utilizou-se o método respondente-driven sampling, considerado adequado para alcançar a população de difícil acesso^13^ e usado também em outros estudos no Brasil.^14^ Esse método envolveu a seleção inicial de “sementes”, pessoas trans com grandes redes sociais que puderam indicar até seis participantes potenciais. As indicadas receberam cupons numerados para rastrear a origem e sequência do recrutamento.

Após a confirmação dos requisitos de elegibilidade e a assinatura do termo de consentimento livre esclarecido, as participantes forneceram materiais biológicos, responderam aos questionários estruturados e passaram por consultas de enfermagem e médicas. Foram utilizados os seguintes formulários para coleta de dados e avaliação clínica: aceitabilidade de coleta e de procedimentos pré-consulta, aceitabilidade de coleta e de procedimentos pós-consulta, avaliação clínica e seguimento e avaliação laboratorial. Com base nos resultados dos exames, as participantes receberam prescrição de tratamentos e vacinas disponíveis no Sistema Único de Saúde conforme recomendação do PNI e histórico de ISTs e tratamentos pregressos. Uma “navegadora de pares”, também mulher trans, auxiliou no acompanhamento das participantes, especialmente no processo de vacinação. A maioria das participantes não era matriculada no Centro de Referência e Treinamento em DST/Aids, local da pesquisa, e recebeu um número identificador para facilitar o registro e acompanhamento em futuras visitas. 

Ao final da sua participação na pesquisa, cada voluntária recebeu ajuda de custo para ressarcir seus gastos com alimentação e com transporte até o local do estudo.

Para caracterização das variáveis socioeconômicas e demográficas deste estudo, foram analisadas as seguintes variáveis independentes: idade, dividida em grupos etários (em anos: 18-19, 20-29 anos, 30-39, ≥40); situação de trabalho (desempregada, empregada com carteira assinada, trabalha por conta própria, empregada sem carteira assinada, realiza trabalhos esporádicos/bicos, aposentadas/em benefícios), principal ocupação ser profissional do sexo (sim, não); escolaridade (em anos: ≤8, 9-12, ≥13); situação de moradia (em casa ou apartamento alugado/próprio, situação de moradia instável [abrigo/instituição/situação de rua ou sem endereço fixo, em uma pensão/casa de cafetina/ocupação ou invasões], mora provisoriamente com família/amigos); religião (não tem religião, católica, afro brasileira [umbanda, candomblé], evangélica/protestante, espírita, orientais [budismo/hinduísmo], judaica, recusou-se a responder), já fez sexo em troca de dinheiro, bens, drogas ou um local para morar (sim, não, não respondeu), identidade de gênero (mulher transexual, travesti, mulher, andrógeno/gênero ambíguo/não binário) e orientação sexual (heterossexual, bissexual, pansexual, homossexual/*gay* ou lésbica, não sei, assexual).

As variáveis dependentes foram adesão e completude dos esquemas vacinais propostos. Adesão foi categorizada em “sim”, “não” e “não tenho certeza”. Completude (operacionalizada em “dose recebida”) foi categorizada em “sim” e “não”.

Para o cálculo das proporções de adesão às vacinas, utilizaram-se os dados do TransOdara disponíveis no Research Eletronic Data Capture (REDCap), ^15^ plataforma de coleta e gerenciamento de dados da pesquisa. Esses dados foram obtidos a partir do preenchimento do formulário de aceitabilidade de procedimentos pós-consulta, no qual constava a pergunta sobre o interesse em receber as vacinas contra HPV e hepatites A e B. Em caso de indicação, foram explicadas a quantidade de doses para cada imunizante, o local de aplicação e a necessidade de retornar ao serviço para completar o esquema indicado.

Os indicadores selecionados para mensurar as proporções de adesão às vacinas de interesse foram: proporção de participantes que aceitaram ser vacinadas caso houvesse indicação; proporção de participantes que aceitaram receber uma injeção no braço na data de atendimento e, após esta, outra vacina entre um mês e seis meses; e proporção de participantes que teriam a disponibilidade de retornar para receber as doses subsequentes das vacinas recomendadas. Para essas duas últimas perguntas, duas participantes não responderam quando foram entrevistadas.

Consideraram-se os motivos relatados a partir de questões abertas no questionário para avaliação da recusa de quaisquer das vacinas ofertadas. Calculou-se o percentual das afirmações mais frequentes.

Para avaliar a completude, a enfermeira da equipe da sala de vacina e da pesquisa acessou o prontuário eletrônico para verificar o retorno das participantes para as doses das vacinas. As informações foram registradas no REDCap para compor o banco de dados da pesquisa. As proporções de completude das vacinas foram calculadas com base na proporção de participantes que receberam as doses recomendadas: vacina contra HPV (1ª, 2ª e 3ª doses), vacina contra hepatite A (1ª e 2ª doses) e vacina contra hepatite B (1ª, 2ª e 3ª doses), considerando apenas aquelas com indicação para cada vacina.

As recomendações para os imunizantes seguiam os critérios do calendário vacinal do PNI, levando em conta o histórico de imunização, idade, prática sexual e *status* sorológico de HIV. Isso explicou a variação no número de participantes indicados para cada tipo de imunizante. O período de recrutamento foi concluído em outubro de 2020, mas foram considerados os retornos à sala de vacina até agosto de 2021. 

No esquema vacinal contra hepatite A, cinco participantes foram encaminhadas para iniciar a vacinação. Três não receberam a primeira dose por motivos não informados. Duas não iniciaram o esquema, porque, quando chegaram à sala de vacinação, verificou-se que já havia registro de doses recebidas para esse imunizante antes do estudo.

Para a vacina contra hepatite B, três participantes foram encaminhadas, mas duas desistiram sem justificativa ao comparecer à sala de vacinas. Uma não iniciou o esquema, porque já havia completado anteriormente, conforme registro obtido no prontuário eletrônico do serviço. Uma participante começou a vacinação, mas, ao retornar para a 2ª dose, constatou-se que ela também já havia completado o esquema antes do estudo.

Durante a pandemia de covid-19, o recrutamento de participantes para a pesquisa no município de São Paulo em um serviço de saúde de referência para HIV/aids e ISTs, que não fechou durante a pandemia, foi interrompido ao atingir metade da amostra planejada. A pausa de cerca de quatro meses foi necessária para respeitar as medidas restritivas, mas não comprometeu o alcance da amostra inicialmente prevista, que foi retomada posteriormente.

As variáveis foram descritas por meio de frequências absolutas e relativas, estratificadas como já mencionado, e o intervalo de confiança (IC_95_%) da proporção de completude foi calculado para cada imunizante. Os pesos *respondente-driven sampling* não foram usados na análise do TransOdara, pois existiam estudos que contraindicavam sua aplicação.^16^ Todas as análises foram realizadas no Stata 14.1. 

A pesquisa foi aprovada pelo Comitê de Ética em Pesquisa da Santa Casa de Misericórdia de São Paulo por meio do parecer n° 3.126.815 de 30/01/2019; certificado de apresentação de apreciação ética 05585518.7.0000.5479.

## RESULTADOS

No estudo TransOdara em São Paulo, participaram 403 mulheres trans e travestis, com idade média de 33,9 anos. Do total, 38,0% tinham entre 20 e 29 anos, 25,0% estavam desempregadas e 21,6% se identificavam como profissionais do sexo, enquanto 50,2% já haviam trocado sexo por dinheiro, drogas, moradia ou outros bens. A maioria (69%) possuía entre 9 e 12 anos de estudo, e 72% moravam em casa ou apartamento próprio ou alugado. Não tinham nenhuma religião 37% das participantes. A maioria das participantes (66,3%) se identificava como mulheres trans, e 85,6% tinham orientação heterossexual (Tabela 1).

**Tabela 1:** Características socioeconômicos e demográficas das participantes do Projeto TransOdara, município de São Paulo, 2019-2020 (n=403)

Variáveis	n (%)
**Faixa etária (anos)**	
18-19	7 (1,7)
20-29	153 (38,0)
30-39	126 (31,3)
≥40	117 (29,0)
**Situação de trabalho** ^a^	
Desempregada	69 (25,0)
Empregada com carteira assinada	62 (22,5)
Trabalha por conta própria	55 (19,9)
Empregada sem carteira assinada	41 (14,8)
Realiza trabalhos esporádicos/bicos	39 (14,1)
Aposentada/em benefício	10 (3,6)
**Principal ocupação profissional do sexo**	
Sim	87 (21,6)
Não	316 (78,4)
**Escolaridade (anos)**	
≤8	67 (16,6)
9-12	280 (69.5)
≥13	56 (13,9)
**Situação de moradia**	
Casa ou apartamento alugado/próprio	291 (72,2)
Situação instável	64 (15,9)
Mora provisoriamente com família/amigos	48 (11,9)
**Religião**	
Não tem religião	149 (37,1)
Católica	91 (22,5)
Afro-brasileira (umbanda ou candomblé)	68 (16,8)
Evangélica/protestante	52 (12,9)
Espírita	35 (8,7)
Orientais/budismo/hinduísmo	4 (1,0)
Judaica	2 (0,5)
Recusou-se a responder	2 (0,5)
**J** **á fez sexo em troca de dinheiro**	
Não	90 (22,3)
Sim	203 (50,2)
Não respondeu	111 (27,5)
**Identidade de gênero**	
Mulher transexual	267 (66,3)
Travesti	95 (23,6)
Mulher	39 (9,7)
Andrógeno/gênero ambíguo/não binário	2 (0,4)
**Orientação sexual**	
Heterossexual	346 (85,6)
Bissexual	22 (3,4)
Pansexual	17 (4,2)
Homossexual, *gay* ou lésbica	16 (4,0)
Não sei	2 (0,5)
Assexual	1 (0,2)

a) Cento e vinte e sete participantes não responderam qual a situação de trabalho no momento em que responderam ao questionário.

Tiveram interesse em receber as vacinas indicadas 88,8% das participantes. Destas, 96,6% referiram que aceitariam receber uma injeção intramuscular no braço na mesma data de atendimento e, após esta, outra vacina entre um mês e seis meses. Referiram também que teriam disponibilidade em retornar e receber as doses remanescentes (Tabela 2). 

**Tabela 2:** Interesse prévio em receber vacinas ofertadas por via intramuscular e completar os esquemas vacinais indicados, município de São Paulo, 2019-2020 (n=403)

Interesse	Sim (%)	Não (%)	Não tem certeza (%)	Total
Aceitabilidade em receber as vacinas indicadas	358 (88,8)	44 (10,9)	1 (0,2)	403
Aceitabilidade em receber injeção no braço na data do atendimento na pesquisa e, após esta, outra vacina entre um mês e seis meses	346 (96,6)	05 (1,4)	5 (1,4)	356^a^
Disponibilidade em retornar para receber as vacinas subsequentes	346 (96,6)	05 (1,4)	5 (1,4)	356^a^

a) 2 participantes não responderam aos questionamentos sobre aceitabilidade prévia em receber injeção no braço na data do atendimento na pesquisa e, após esta, outra vacina entre um mês e seis meses e disponibilidade em retornar para receber as vacinas subsequentes.

A oferta de encaminhamento à sala de vacina foi recusada por 44 participantes, independentemente do esquema vacinal indicado. Os motivos para a recusa também foram levantados, sendo os mais comuns referidas pelas participantes: estar com a carteira de vacinação atualizada ou completa, ter medo/pavor de agulhas, estar com pressa ou preferir receber a vacina em outro serviço (Tabela 3). 

**Tabela 3:** Motivos apresentados pelas participantes para justificar a recusa em receber as doses de vacinas indicadas pelos profissionais do projeto TransOdara, município de São Paulo, 2019-2020 (n= 44)

Motivo da recusa em receber vacinas indicadas	n
Carteira de vacinação completa	27
Medo de agulha	7
Pressa	3
Preferência por outro serviço	3
Ausência de interesse	1
Residência distante do local que recebeu a primeira dose	1
Aversão a uso de medicamentos	1
Preferência por avaliação por infectologista	1

Observou-se que 77,2% (IC_95_% 71,7;82,1) receberam a primeira dose contra hepatite A, 70,9% (IC_95_% 64,0;77,2) contra hepatite B e 88,2% (IC_95_% 79,4;94,2) contra HPV. A proporção de recusa da primeira dose da vacina contra HPV (11,8%, IC_95_% 5,8;20,6) foi significativamente menor que aquela observada para a vacina contra hepatite B (27,5%, IC_95_% 21,4;34,4). As proporções de completude para os esquemas vacinais foram 12,0% (IC_95_% 8,0;17,3) para vacina contra hepatite A, 7,2% (IC_95_%, 3,5;12,8) contra hepatite B e 8,1% (IC_95_% 3,0; 16,6) contra HPV, não sendo observada diferença estatisticamente significativa entre elas.

Algumas participantes que aceitaram receber os esquemas vacinais indicados, quando chegaram a sala de vacina, recusaram a aplicação, sendo 20,8% na vacina contra hepatite A, 27,5%, hepatite B e 11,8% para as vacinas HPV (Tabela 4).

**Tabela 4:** Indicação, número e intervalo de confiança de 95% (IC_95_%) de doses recebidas das vacinas contra hepatite A, hepatite B e papilomavírus humano (human papillomavirus, HPV) pelas participantes, município de São Paulo, 2019-2021

Dose	Hepatite A (n=268)		Hepatite B (n=196)		HPV (n=85)	
	n	% (IC _95_ % )	n	% (IC _95_ % )	n	% (IC _95_ % )
Primeira						
Aceitou	207	77,2 (71,7;82,1)	139	70,9 (64,0;77,2)	75	88,2 (79,4;94,2)
Recusou	56	20,8 (16,2;26,3)	54	27,5 (21,4;34,4)	10	11,8 (5,8;20,6)
Outra situação	5	1,9 (0,6;4,3)	3	1,5 (0,3;4,4)	-	-
**Segunda**						
Realizou	25	12,0 (8,0;17,3)	19	13,7 (8,4;20,5)	10	13,3 (6,6;23,2)
Não compareceu	182	86,1 (82,7;92,0)	119	85,6 (78,7;91,0)	65	86,7 (76,8;93,4)
Outra situação	-	-	1	0,7 (0,01;3,9)	-	-
**Terceira**						
Realizou	-	-	10	7,2 (3,5;12,8)	6	8,1 (3,0;16,6)
Não compareceu	-	-	9	6,5 (3,0;11,9)	4	5,3 (1,5;13,1)
Outra situação	-	-	-	-	-	-

## DISCUSSÃO

Observou-se a alta adesão às vacinas contra HPV e hepatites A e B, mas a baixa completude dos esquemas vacinais, mesmo após a equipe informar os indivíduos sobre os benefícios, a via de administração e a periodicidade de cada vacina. A falta de estudos específicos sobre o uso de vacinas por mulheres trans e travestis como prevenção de ISTs dificulta a comparação direta dos resultados. Os dados deste estudo foram comparados com os de homens que fazem sexo com homens, pessoas vivendo com HIV e a população em geral, especialmente em relação à completude da vacinação contra hepatite A.

A análise da aceitação da vacina contra hepatite A entre diferentes populações pode revelar variáveis que influenciam a adesão à imunização. Neste estudo, a proporção de aceitação observada foi superior à registrada entre homens que fazem sexo com homens em Victoria, Austrália, que foi 62,7%. Tal aceitação foi medida pelo recebimento de pelo menos uma dose da vacina.^17^ Nesta pesquisa foi considerada a manifestação de interesse em receber a vacina como critério de aceitação.

As populações estudadas apresentaram semelhanças significativas em relação às vulnerabilidades sexuais e recomendações sobre vacinas, mas diferiram nas proporções de adesão. Essa discrepância pôde ser explicada pela diferença na idade média dos participantes: 33,9 anos neste estudo contra 29 anos em Victoria. Isso refletiu níveis variados de conscientização sobre imunização e prioridades de saúde. A vacinação foi oferecida com base no *status* sorológico prévio,^17^ ao passo que este estudo buscou o interesse em receber o imunizante de todas as participantes.

A adesão à vacina contra HPV neste estudo foi 88,8%, resultado positivo que indicou a intenção das participantes em utilizar a vacina como estratégia de prevenção de ISTs. Esse dado foi próximo á proporção de 87,0% encontrada na Cidade do México, que incluiu população adulta, com 9,3% de mulheres trans.^18^ Valor semelhante de 90,0% foi observado em profissionais do sexo de Teresina, Piauí,^19^ para a vacina contra hepatite B. As altas proporções de adesão aos esquemas vacinais contra HPV e hepatite B pareceram estar relacionadas à maturidade das participantes, cuja idade média variou entre 31,9 e 33,9 anos. Isso sugeriu que a maturidade pode influenciar na conscientização sobre saúde sexual e a aceitação das vacinas, além de promover a valorização das oportunidades de prevenção e cuidado com a saúde. 

Considerando o imunizante contra hepatite B, a adesão deste estudo foi superior à observada em Paris, que incluiu populações de risco como homens que fazem sexo com homens, pessoas que usam drogas injetáveis e mulheres trans. Entre os elegíveis que não foram vacinados nem infectados, a aceitabilidade para receber a primeira dose foi 53,0% nas mulheres trans.^20^

As populações de difícil acesso, mulheres trans e travestis, profissionais do sexo e homens que fazem sexo com homens apresentaram maior vulnerabilidade relacionada às práticas sexuais e, consequentemente, maior risco para contrair ISTs curáveis. Em função disso, não basta ter os imunizantes disponíveis nos serviços de saúde que atendam esses grupos populacionais. Para ampliar as proporções de adesão para os referidos esquemas é necessário incluir estratégia como a busca ativa. 

A completude do esquema vacinal contra HPV neste estudo apresentou resultados intermediários em comparação com uma amostra de homens que fazem sexo com homens e mulheres trans de duas cidades dos Estados Unidos, onde 4,6% relataram ter recebido as três doses, principalmente em serviços privados.^21^ Em contraste, uma pequena amostra nacional de jovens adultos identificados como trans nos Estados Unidos mostrou a completude superior a 60%.^22^ As semelhanças nas características relacionadas à indicação, à faixa etária e ao alto grau de escolaridade não foram suficientes para assegurar que as participantes desta amostra atingissem a completude do esquema deste imunizante. As barreiras econômicas, destacadas nos estudos desenvolvidos naquele país, evidenciam que o maior acesso ocorreu em clínicas privadas e que há falta de incentivo por parte dos profissionais de saúde. Na amostra deste estudo, a vacinação foi oferecida no serviço público. A baixa completude pôde estar relacionada à dificuldade financeira para custear as passagens de transporte público e retornar ao serviço e completar o esquema vacinal, já que 25% da população declarou estar desempregada no momento do recrutamento. 

A oferta da vacina contra hepatite A não tem garantido altas proporções de completude do esquema vacinal, mesmo quando gratuita para pessoas vivendo com HIV no Brasil e em uma pequena proporção de países da Europa central e ocidental.^23^ Na França, 54,2% das pessoas vivendo com HIV recomendadas para receber a vacina foram imunizadas de fato.^24^ No Reino Unido, a vacina também é gratuita e indicada para adultos com alto risco, incluindo homens que fazem sexo com homens. Considerando a população geral britânica, a completude do esquema vacinal para hepatite A alcançou 11%.^25^

A baixa completude do esquema vacinal contra hepatite B foi observada em Paris, aonde nenhuma participante retornou para a terceira dose.^20^ Em Teresina, as profissionais do sexo atingiram a completude de 26,3%, devido a estratégia de agendamento adotada, que buscava adequar a disponibilidade da participante para receber o imunizante.^19^ Estratégia semelhante de agendamento foi adotada em Goiânia, atingindo completude de 55,3% e 41,5% para dois esquemas diferentes (um convencional de três doses e um superacelerado de quatro doses).^26^ Sugeriu-se que esquemas vacinais mais curtos podem melhorar a adesão, especialmente em populações de difícil acesso.

A pandemia de covid-19 teve impacto significativo na completude dos esquemas vacinais especialmente entre populações vulneráveis como as minorias sexuais, tanto por questões financeiras como pelo distanciamento proposto pelo governo do estado.^27^ Recrutamento *online* de indivíduos trans no Brasil evidenciou que 59,0% relataram alto impacto das medidas de distanciamento social, com 54,7% indicando que a situação econômica foi a mais afetada.^28^ Os principais desafios enfrentados foram a manutenção do emprego e a redução salarial. Mesmo com a continuidade do serviço de vacinação, a de completude dos esquemas vacinais sofreu impacto negativo. 

A estratégia de navegadores de pares mostrou-se eficaz no aumento da adesão às vacinas, promovendo autocuidado e melhorando o envolvimento com os tratamentos, especialmente entre grupos vulneráveis. Esse modelo buscou facilitar o acesso a serviços de saúde e disseminar conhecimento sobre práticas preventivas contra ISTs. A efetividade dessa abordagem foi observada em São Paulo com 94,4% das participantes recomendando a estratégia de navegadores de pares para a adesão ao tratamento antirretroviral entre pessoas vivendo com HIV/aids.^29^

Este estudo não contou com a presença de navegadora de pares para acompanhar as participantes nas visitas subsequentes à sala de vacinas, com recursos financeiros para cobrir os custos de transporte das participantes para o retorno ao serviço e com estratégias de busca ativa para localizar aquelas que faltaram às doses subsequentes. Não foi possível verificar se as participantes receberam doses adicionais em outros serviços de saúde do Sistema Único de Saúde. 

Este estudo demonstrou potencialidades significativas, como o cumprimento da amostra prevista, mesmo com a interrupção causada pela pandemia de covid-19. As participantes expressaram satisfação em ter acesso às vacinas recomendadas para a prevenção de infecções sexualmente transmissíveis.

Apesar da identificação de alta adesão para receber os imunizantes, a baixa completude deve chamar a atenção dos formuladores de políticas de imunização. Considerando-se as barreiras que as pessoas trans enfrentam em acessar serviços de saúde e os riscos aumentados para ISTs imunopreviníveis,^30^ faz-se necessário sensibilizar os profissionais de saúde que atendem essa população. Inovar em estratégias que resultem na ampliação do acesso aos serviços também é fundamental, implementando novas metodologias assistenciais que favoreçam a inclusão dessa população nas rotinas dos serviços de saúde.

Um dos principais desafios da saúde é promover a educação em saúde contínua para profissionais que atendem pessoas trans, visando sensibilizá-los sobre as realidades e condições de vida dessas pessoas. Isso é essencial para reduzir resistências, ampliar o acesso e garantir o atendimento digno e respeitoso. Faz-se crucial desenvolver iniciativas educativas para a população trans, incentivando a adesão e conclusão dos esquemas vacinais recomendados. O esforço conjunto dos órgãos de saúde é necessário para aumentar a oferta de imunizantes para a prevenção contra as ISTs e dos profissionais de saúde para informar e conscientizar sobre essas formas de prevenção.
